# How to Build a Low-Cost Video-Assisted Laryngoscopy Suite for Airway Management Training

**DOI:** 10.21980/J8C068

**Published:** 2023-04-30

**Authors:** Erin Falk, Adam Blumenberg

**Affiliations:** * Columbia University Medical Center, Department of Emergency Medicine, New York, NY; ^ Poison Center of Oregon, Alaska, and Guam. Portland, OR

## Abstract

**Audience:**

This suite of borescope laryngoscopes is designed to instruct emergency medicine residents and sub-interns in video-assisted airway management.

**Background:**

Skillful and confident airway management is one of the markers of a strong emergency medicine physician.^1^ Video-assisted airway management is a necessary skill, particularly in the setting of difficult airways and cervical spine immobilization.^2^,^3^ However, the idea of learning airway management “by doing” is high-risk and mistakes can have devastating implications on patient outcomes. Fortunately, high-fidelity medical simulation tools have been developed to address this dilemma, allowing a safe environment for providers to practice their airway management skills.^4^,^5^ These tools, while undeniably useful, are limited in their scope; they are often designed for clinical rather than educational use, and are proprietary and expensive.^6^,^7^

Video laryngoscopes approved for patient use are difficult to implement widely in educational settings due to cost or because they cannot be removed from a designated area. Clinical video laryngoscopy suites typically cost 2,000 – 6,000 US dollars. Additionally, the video images can only be viewed on a local small screen rather than a television or projector. This means that the number of learners is limited by space around the small laryngoscope screen. These cost and space barriers may be especially pronounced in low resource or non-traditional learning environments.

**Educational Objectives:**

Using an anatomically accurate airway simulator, by the end of a 20–30-minute instructional session, learners should be able to: 1) Understand proper positioning and use the video laryngoscope with dexterity, 2) identify airway landmarks via the video screen, and 3) demonstrate ability to intubate a simulated airway.

**Educational Methods:**

We developed a low-cost borescope laryngoscope for airway simulation training. Using this device, learners should be able to identify airway landmarks and successfully intubate a simulated airway. The borescope laryngoscope, a novel device which employs the camera-end of a video borescope and a single-use VL blade, was used by learners during high-fidelity airway simulation. Learners were residents or medical students undergoing airway training in case-based simulation, or in airway-management procedure stations.

**Research Methods:**

The borescope laryngoscopes were used during dedicated airway training in place of their medical device counterparts. During case-based simulation sessions involving airway management, 32 residents and 20 medical students used the borescope laryngoscope. During dedicated airway management procedure stations, 12 medical students used the borescope laryngoscope. Learners were instructed to perform endotracheal intubation and fully visualize critical structures before passing the tube. Successful intubation was defined as the ability to pass the tube independently or with the help of the instructor.

**Results:**

The borescope laryngoscope proved effective at video visualization of critical structures. Compared to official medical equipment, the VL borescope similarly allowed for visualization of a Cormack-Lehane Grade 1 view. Learners were able to visualize the airway anatomy and successfully pass the ET tube on each pass either independently or with the help of the instructor.

**Discussion:**

The development of this airway-training tool was effective and less expensive than medical grade versions. Our group of learners successfully visualized essential anatomy and passed an endotracheal tube (ED tube) through the vocal cords. The borescope laryngoscope offers a comparable user experience at a much lower cost. The devices also allowed instructors to teach video laryngoscopy without depending on clinical equipment. Widespread use may allow for expansion of airway simulation training while maintaining a high-fidelity learner experience.

**Topics:**

Video laryngoscopy, borescope, improvised equipment, airway training.

## USER GUIDE

**Table t1-jetem-8-2-i1:** 

List of Resources:	
Abstract	1
User Guide	3

[Table t1-jetem-8-2-i1] **Learner Audience:**

Medical Students, Interns, Junior Residents, Senior Residents

**Time Required for Implementation:**

Building the borescope laryngoscope suite takes approximately one to three hours per borescope laryngoscope device because producing one device may take two hours while producing three devices may take three hours. The learners may spend as much time as needed to improve their video laryngoscopy skills, typically with multiple sessions of 15–20 minutes each.

**Recommended Number of Learners per Instructor:**

The ideal number of learners per instructor is four to six for simulation sessions. If using a USB enabled borescope laryngoscope viewed on a projector or large screen, the number of observers may be increased to 10–20.

**Topics:**

Video laryngoscopy, borescope, improvised equipment, airway training.

**Objectives:**

Using an anatomically accurate airway simulator manikin, by the end of a 20–30 minute instructional session, learners should be able to:

1. Understand proper positioning and use the video laryngoscope with dexterity.
2. Identify airway landmarks via the video screen.
3. Demonstrate ability to intubate a simulated airway.

### Linked objectives and methods

Emergency department residents often practice video laryngoscopy skills with a dedicated airway trainer manikin. Instructors will present trainees with a clinical simulation case and/or airway management scenario and provide training via the borescope laryngoscope. Trainees will appropriately position the airway trainer manikin, handle the borescope laryngoscope as a video laryngoscope, and prepare for intubation (Objective 1).

After appropriately positioning the airway trainer manikin and inserting the borescope laryngoscope into the oropharynx, trainees will identify the vocal folds, arytenoid cartilage, interarytenoid notch, and pharynx (Objective 2).

Once the trainee identifies the relevant anatomic structures of the airway trainer, the trainee will pass an endotracheal tube through the vocal cords. The trainee should then verbalize or perform post-intubation safety procedures such as inflating the endotracheal balloon, confirming tube placement via capnometry and bilateral chest auscultation, and securing the tube (Objective 3).

### Recommended pre-reading for instructor

There are multiple basic and advanced education resources for emergency airway management. The recommended pre-reading for this education modality is “Chapter 29: Tracheal Intubation” of the 9^th^ edition of *Tintinalli’s Emergency Medicine: A Comprehensive Study Guide*.^11^ This textbook chapter includes high quality instruction regarding technique, equipment, positioning, and preparation. The chapter additionally includes high-resolution color photographs which may be adapted for teaching with the borescope laryngoscope.

### Implementation Methods

**Figure 1:**
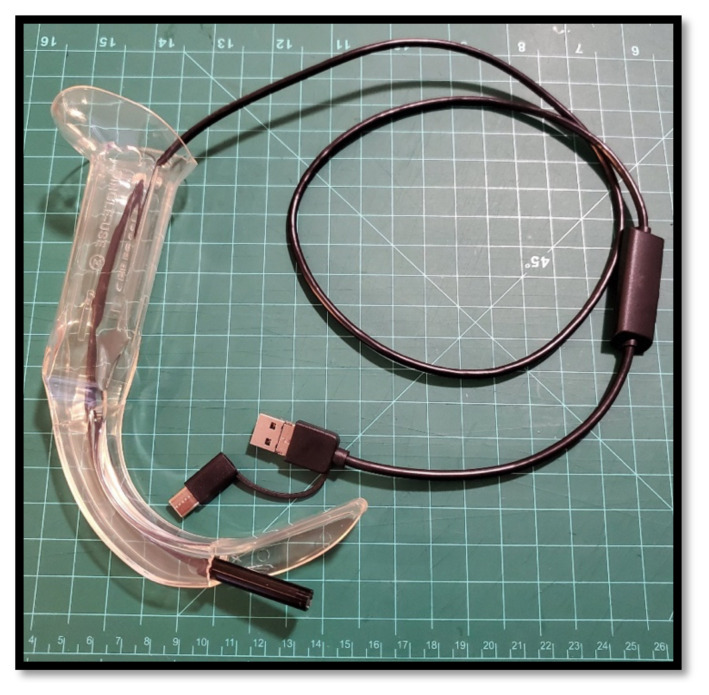
A USB-enabled borescope is placed in a partially completed borescope laryngoscope.

**Figure 2:**
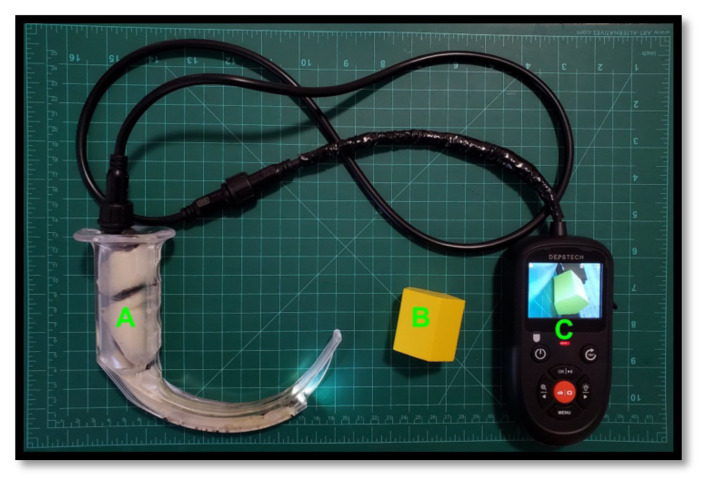
A borescope laryngoscope (A) viewing a yellow block (B) which is displayed on the handheld screen (C).

A low-cost and mobile video laryngoscopy suite that can display images on a computer screen may increase access to video laryngoscopy training.^8^,^9^ Borescopes are small and inexpensive digital video cameras which display live action video via a USB interface or handheld screen. In this innovation, we adapted borescopes to be compatible with existing laryngoscopy equipment. We present the development of a low-cost video laryngoscopy suite for airway training, addressing a clear need for an accessible, affordable, and adaptable airway simulation tool (Figures 1 and 2).

The borescope laryngoscopes require an anatomic airway simulator manikin. These can be used in a simulation laboratory or brought to a hospital setting, ambulance, conference room, classroom, or the field. The educator should provide feedback on airway maneuvers and intubation technique.

**Figure 3:**
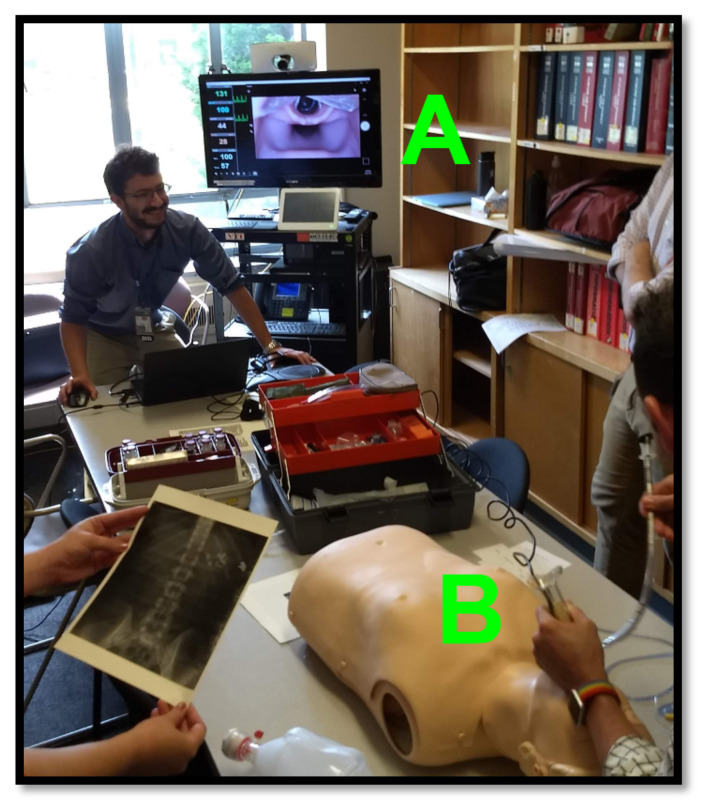
A computer monitor (A) is used so that multiple learners may view the video output of the USB Borescope laryngoscope (B) during a simulation session in a conference room.

If the borescope laryngoscope is USB operated, it will require a computer connection (see Figure 3). The display can be small, such as with a laptop, or large such as with a television screen. This allows several learners to view and learn from the airway procedure simultaneously.^10^ Additionally, a computer connection would allow the educator to record video which may be used during the debrief to review airway maneuvers. If the borescope laryngoscope is connected to an independent display, it may be rested on the stretcher, mounted on a pole, or held by an assistant.

The didactic portion of the airway training should include a brief demonstration by the instructor of appropriate technique in video-assisted laryngoscopy. There are no unique or additional requirements to explaining the borescope laryngoscope to learners; the instructor should demonstrate appropriate intubation technique, use of a rigid stylet in the endotracheal tube, and maneuvering of the endotracheal tube during intubation. The borescope laryngoscope airway training may be a stand-alone activity (as with a procedure station), or may be integrated into a simulation case in which the simulated patient requires intubation. The airway training may be facilitated by one or more instructors and may be as brief as ten minutes or as long as one hour.

### List of items required to replicate this innovation

1. An anatomic airway trainer (eg, Laerdal Airway simulator). Estimated cost $300–$2700.
2. An electric rotary tool (eg, Dremel flex shaft rotary tool). Estimated cost $200.
3. One or more borescopes (eg, DEPSTECH industrial borescope with 4.3” LCD screen, or Sing F Ltd. USB borescope for Android and PC). Estimated cost $20–$60 per unit.
4. One or more transparent single-use video laryngoscope blades (eg, STORZ C Mac single-use scope blade, or Verathon Glidescope single-use blade). Either a standard or a hyper-angulated blade may be used. Estimated cost $10 per unit.
5. Expanding foam glue (eg, Gorilla original formula glue). Estimated cost $8.
6. Fast-setting glue (eg, Krazy glue). Estimated cost $4.
7. (optional) Moldable glue (eg, Sugru moldable multipurpose glue). Estimated cost $12.

### Approximate cost of items to create this innovation

A USB enabled borescope laryngoscope costs approximately 30 US dollars to produce. A mobile, video screen-enabled borescope laryngoscope costs approximately 60 US dollars to produce. Anatomic airway trainer manikins vary in cost from 300 to 3,000 US dollars.

### Detailed methods to construct this innovation

The intended product is to place the camera-end of a video borescope securely within a single-use video laryngoscope blade. The other end can be plugged into a computer by USB to display video, or may connect to a handheld display screen. The final product, the borescope laryngoscope, may be used in place of a clinically-approved laryngoscope in a simulation setting.

**Figure 4:**
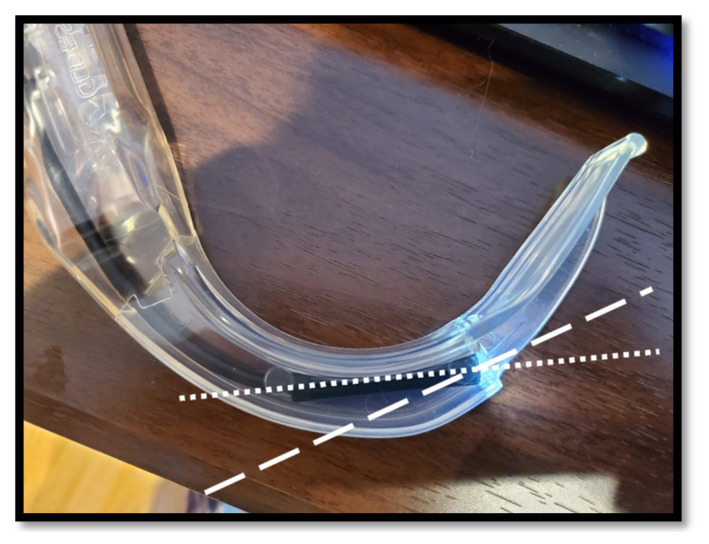
The optical axis of the borescope (dotted line) should as close as possible align with the optical axis of the laryngoscopy blade (dashed line). To improve methods of improving the alignment of the axes see figure 5 and 6.

**Figure 5:**
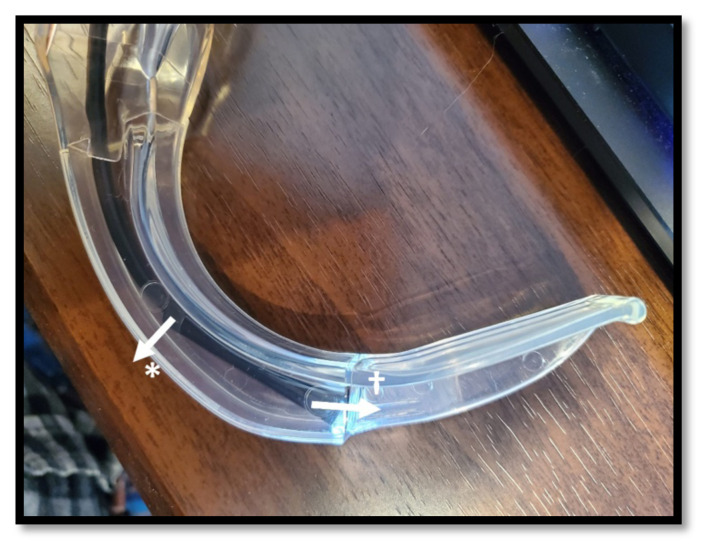
If the optical axes of the borescope and laryngoscope blade to not align easily, use a rotary tool to remove either the concave surface of the blade (*), or the plastic window at distal aspect of the enclosure (†).

**Figure 6:**
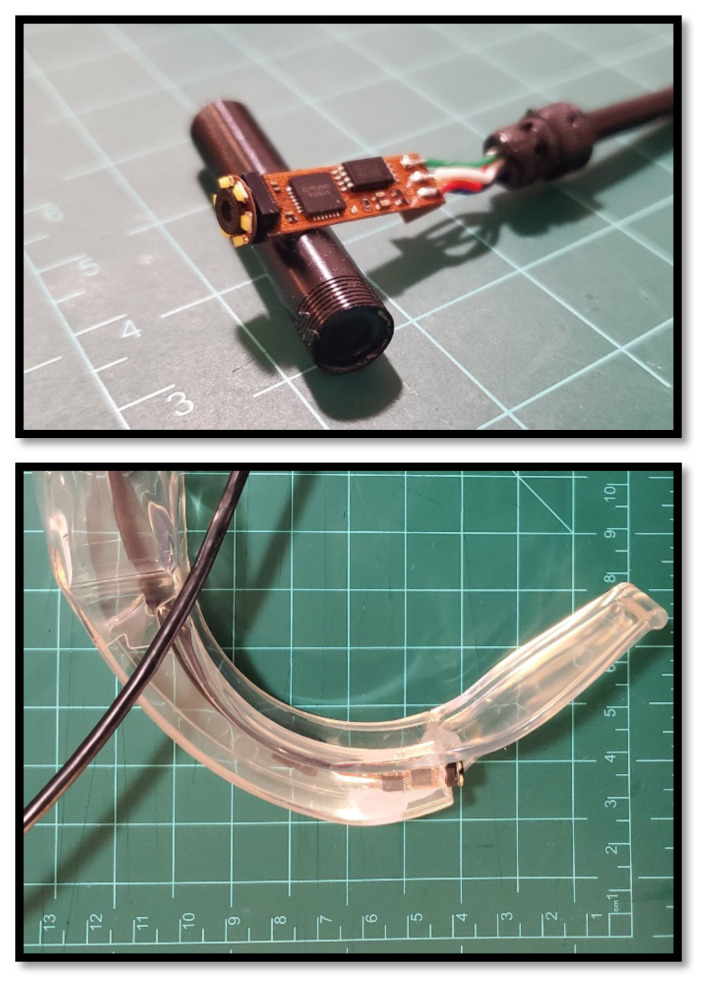
If the optical axes of the borescope and laryngoscope blade to not align easily, the camera end of the borescope is encased in a rigid metal cylinder which can be removed to shorten it (upper image). The borescope may then be placed in the laryngoscopy blade more effectively (lower image).

**Figure 7:**
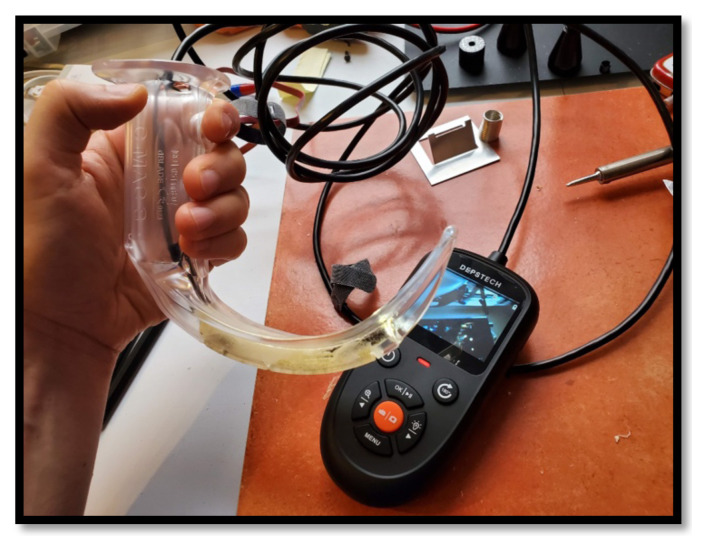
Once the borescope is appropriately positioned and glued within the blade, an expanding foam glue may be used to fill the empty space and stabilize the components.

1. The selected borescope is placed inside the transparent laryngoscope blade (Figure 4). The optical axis of the borescope and blade should be parallel and in line with one another. If the borescope does not fit such that the axes are lined up, one or more of the following techniques can be used to correct this:
  - Using the rotary tool, the convexity of the laryngoscope blade may be removed so that there is more room for the posterior part of the camera (Figure 5, * indicator). Once the camera is securely in place, the defect in the laryngoscope can be patched with moldable glue, which forms a smooth finish.
  - Using the rotary tool, the anterior portion of the laryngoscope blade may be removed so that the camera can be advanced (Figure 5, † indicator). The camera should be advanced as little as possible while still lining up the axes.
  - The borescope camera is often encased within a metal cylinder, which can be removed using pliers. This can be used to shorten the length of the rigid component of the camera and allow the borescope to be further advanced (Figure 6). If this technique is used, care should be taken not to damage the lens or circuit board.
2. The view screen for the borescope (either handheld display or monitor) is used to align the horizon. The laryngoscope blade is placed so that the concavity rests on a flat and level surface and is pointed at a window. The borescope is gently rotated in place until the horizon is appropriately aligned.
3. Once the borescope is correctly positioned and leveled, it is secured in place with a small amount of fast-setting glue. Once secured, expanding foam glue is placed to further secure the borescope within the laryngoscope (Figure 7).

### Results and tips for successful implementation

A suite of borescope laryngoscopes was used at two academic emergency residency programs and medical schools. They were used during two different educational modalities for airway training: 1) simulated case-based scenarios and 2) dedicated procedure training. The borescope laryngoscopes were used during 35 case-based simulation sessions and 2 procedure training sessions to successfully teach intubation and advanced airway skills on anatomic airway models. During case-based simulation sessions involving airway management, 32 residents and 20 medical students used the borescope laryngoscopes. During dedicated airway management procedure stations, 12 medical students used the borescope laryngoscopes. The simulation sessions were a mixture of case-based simulations (primarily toxicology-themed resuscitations), and dedicated airway skills sessions.

A USB-connected borescope laryngoscope was used to facilitate medical simulation sessions in a poison center conference room (see Figure 3). A portable monitor-connected borescope laryngoscope was used to teach airway skills in a simulation laboratory. These tools are easily accessible and portable and may be considered “simulation ready to use” with little preparation time. They are lightweight, small, and easily portable.

**Figure 8:**
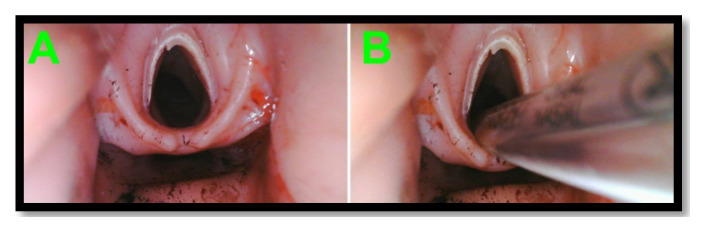
Still images of a simulated bloody airway (A) and successful intubation (B) captured with a borescope laryngoscope.

The borescope laryngoscopes performed well and were sufficiently durable to withstand multiple uses without damage; to date, none have been so damaged as to be non-functional. The field of view and image resolution were adequate for performing intubation (Figure 8). Verbal feedback from learners and educators was universally positive.

The borescope laryngoscope proved effective at video visualization of critical structures. Compared to official medical equipment, the VL borescope similarly allowed for visualization of a Cormack-Lehane Grade 1 view. All learners were able to visualize the airway anatomy and successfully pass the ET tube on each pass either independently or with the help of the instructor. Successful passage was determined by the instructor.

Tips for successful implementation include:

1. A small amount of surgical lubricant on the convex portion of the laryngoscope blade enables better manipulation of the device.
2. If using a USB-connected borescope laryngoscope, ensure the cord is adequately long to connect to the computer. If it is not, a USB extender cord may be used.
3. If using a portable display-connected borescope laryngoscope, spare batteries or power source should be kept with the device.
